# Crystal structure of poly[[di-μ_2_-aqua-aqua­sodium] 4-amino-3,5,6-tri­chloro­pyridine-2-carboxyl­ate trihydrate], the sodium salt of the herbicide picloram

**DOI:** 10.1107/S2056989015012633

**Published:** 2015-07-15

**Authors:** Graham Smith

**Affiliations:** a Science and Engineering Faculty, Queensland University of Technology, GPO Box 2434, Brisbane, Queensland 4001, Australia

**Keywords:** crystal structure, picloram, Tordon, herbicide, sodium salt, coordination polymer, sodium–water cationic chain, hydrogen bonding

## Abstract

In the structure of the title complex, {[Na(H_2_O)_3_](C_6_H_2_Cl_3_N_2_O_2_)·3H_2_O}_*n*_, the sodium salt of the herbicide picloram, the cation adopts a polymeric chain structure, based on μ_2_-aqua-bridged NaO_5_ trigonal–bipyramidal complex units which have, in addition, a singly bonded water mol­ecule. Each of the bridges within the chain, which extends parallel to the *a* axis, is centrosymmetric, with Na⋯Na separations of 3.4807 (16) and 3.5109 (16) Å. In the crystal, there are three water mol­ecules of solvation and these, as well as the coordinating water mol­ecules and the amino group of the 4-amino-3,5,6-tri­chloro­picolinate anion, are involved in extensive inter-species hydrogen-bonding inter­actions with carboxyl and water O atoms, as well as the pyridine N atom. Among these associations is a centrosymmetric cyclic tetra­water *R*_4_^4^(8) motif, resulting in an overall three-dimensional structure.

## Abstract 2

The trihydrated sodium salt of the herbicide picloram comprises a cationic μ_2_-aqua-bridged chain structure that is linked to the picloramate anions through amine N—H⋯O and water O—H⋯O and O—H⋯N hydrogen bonds.

## Chemical context   

4-Amino-3,5,6-tri­chloro­pyridine-2-carb­oxy­lic acid (picloram) is a commercial herbicide (Mullinson, 1985 ▸) introduced by Dow Chemicals as Tordon (O’Neil, 2001 ▸). Although it has potential as a metal-chelating ligand similar to picolinic acid, there are only five metal complexes with picloramate anions in the crystallographic literature. Examples include picloram as a bidentate *N,O* chelating ligand with Mn^II^ (Smith *et al.*, 1981*a* ▸) and Cu^II^ (two structures, one a mixed-ligand complex with 2-amino­pyrimidine; O’Reilly *et al.*, 1983 ▸) and caesium (Smith, 2013 ▸). In the Mg complex (Smith *et al.*, 1981*b* ▸), the picloramate anions act as counter-ions to the [Mg(H_2_O)_6_]^2+^ cation. Although the structure of picloram has not been reported, that of the guanidinium salt is known (Parthasarathi *et al.*, 1982 ▸). The reaction of picloram with sodium bicarbonate in aqueous ethanol gave crystals of the title complex salt {[Na(H_2_O)_3_]^+^·C_6_H_2_Cl_3_N_2_O_2_^−^·3H_2_O}_*n*_, and the structure is reported herein.
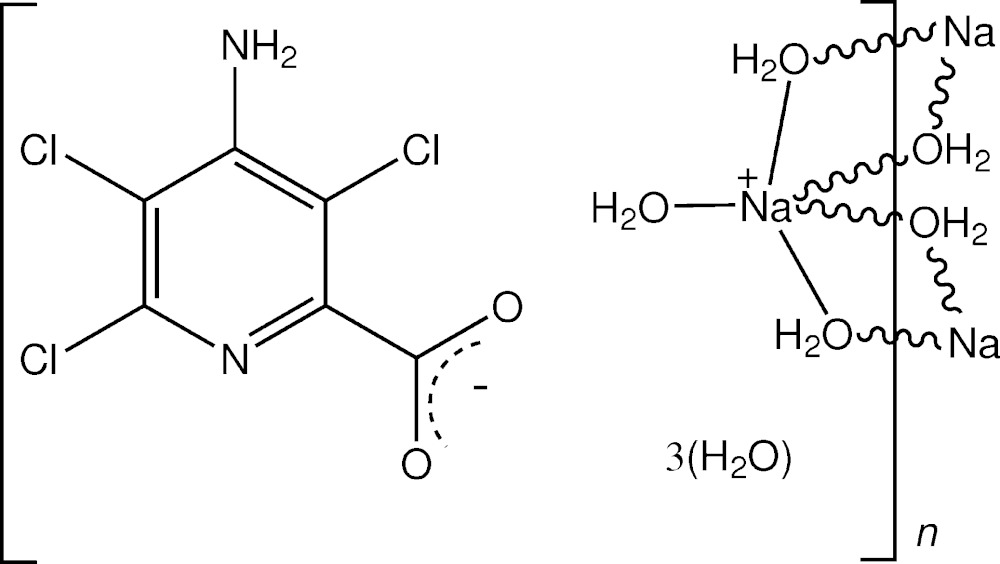


## Structural commentary   

**Figure 1:**
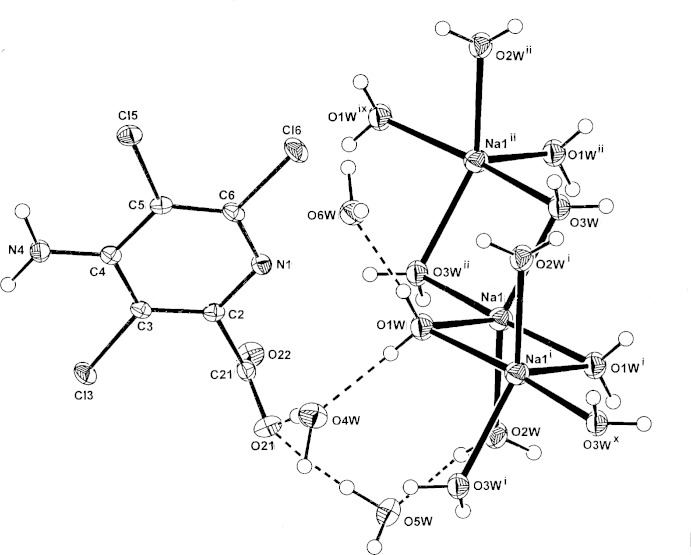
The atom-numbering scheme for the hydrated title salt, with non-H atoms drawn as 40% probability ellipsoids. Inter-species hydrogen bonds are shown as dashed lines. Symmetry codes: (ix) *x* + 1, *y*, *z*; (x) *x* − 1, *y*, *z*. For other symmetry codes, see Table 1 ▸.

In the structure of the title salt, (Fig. 1 ▸), polymeric cationic chains based on μ_2_-water-bridged NaO_5_ trigonal–bipyramidal complex units are formed, comprising centrosymmetric four-membered water-bridged Na_2_O_2_ rings with both O1*W* and O3*W* [Na⋯Na^i^ and Na⋯Na^ii^ = 3.4807 (16) and 3.5109 (16) Å, respectively; for symmetry codes, see Table 1 ▸]. In the fifth Na coordination site is the third water mol­ecule (O2*W*) in a non-bridging mode [overall Na—O range, 2.3183 (17)–2.4185 (16) Å: Table 1 ▸]. Although the μ_2_-water-bridged cationic chains are relatively common, the NaO_5_ coordination with one non-bridging water is rare, compared to the more usual octa­hedral NaO_6_ coordination involving two non-bridging water mol­ecules in other examples, *e.g.* in the biphenyl-4,4′-di­phospho­nate salt (Kinnibrugh *et al.*, 2012 ▸).

The structure of the title salt also contains non-coordinating picloramate anions and three water mol­ecules of solvation (O4*W*–O6*W*). In this anion, the carboxyl group lies close to perpendicular to the pyridine ring [torsion angle N1—C2—C21—O21 = 89.1 (2)°], which is similar to that in the anhydrous guanidinium picloramate salt (73.3°) (Parthasarathi *et al.*, 1982 ▸), while the amine group gives lateral intra­molecular N4—H41⋯Cl3 and N4—H41⋯O6*W* inter­actions [2.9956 (17), 3.080 (2) Å].

## Supra­molecular features   

**Figure 2:**
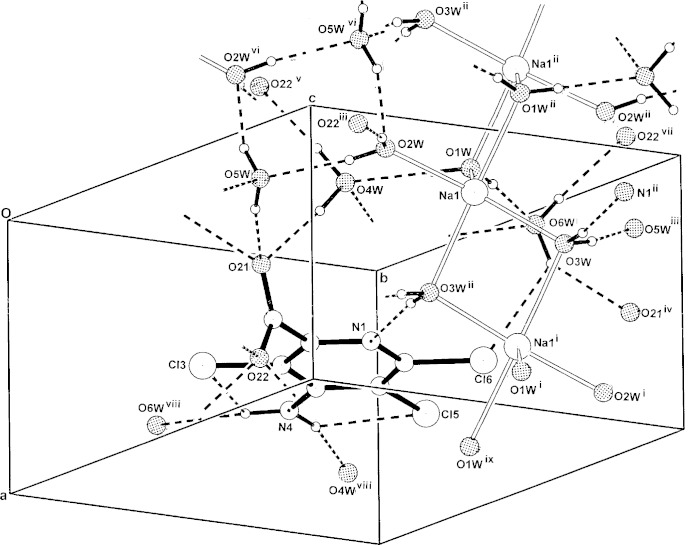
The three-dimensional hydrogen-bonded structure, with inter-species hydrogen bonds and intra­molecular N—H⋯Cl associations shown as dashed lines. For symmetry codes, see Fig. 1 ▸ and Table 2 ▸.

In the crystal there are numerous inter-species water O—H⋯O_carboxyl,water_, O—H⋯N_pyridine_ and O—H⋯Cl hydrogen-bonding inter­actions (Table 2 ▸), including a centrosymmetric tetra-water cyclic ring involving O2*W*—H⋯O5*W* and O5*W*—H⋯O2*W* ^vi^ [graph set 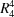
(8)], giving a three-dimensional structure (Fig. 2 ▸). Cyclic tetra-water moieties such as found in the present structure are being identified in an increasing number in labile water-stabilized salt hydrates, *e.g.* in the brucinium l-glycerate 4.75-hydrate salt (Białońska *et al.*, 2005 ▸). Also found in the structure of the title salt is a short inter­molecular Cl3⋯Cl5^xi^ contact [3.2108 (7) Å; symmetry code (xi): *x*, *y* − 1, *z*].

## Database survey   

The (μ_2_-aqua)-bridged Na_2_(H_2_O)_2_ units in the coordination polymeric cationic chains in the title structure have precedents in a large number of reported crystal structures. However, with few exceptions, these are based on NaO_6_ polyhedra, with octa­hedral or distorted octa­hedral stereochemistry, having two non-bridging water mol­ecules [Na_2_(H_2_O)_8_^2+^], compared to one non-bridging water mol­ecule in the NaO_5_ coordination [Na_2_(H_2_O)_6_^2+^] of the title complex. The [Na_2_(H_2_O)_8_^2+^] dicat­ions may be discrete, such as found in the anionic aryl­telluronic anhydride salt (Beckmann *et al.*, 2012 ▸) and the anionic di­methyl­arsenate (cacodylate) salt (Lennartson & Håkansson, 2008 ▸), or they may be found as [Na_4_(H_2_O)_16_^4+^] tetra-cations as found in the dianionic biphenyl-4,4′-di­phospho­nate salt (Kinnibrugh *et al.*, 2012 ▸) and the monoanionic salt of luminol (5-amino-2,3-di­hydro-1,4-phthal­azinedione; Guzei *et al.*, 2013 ▸). However, more commonly, they are polymeric [Na_2_(H_2_O)_8_]_*n*_, *e.g.* in the monoanionic salt of the anti-allergic drug tranilast ({2-[3-(3,4-di­meth­oxy­phen­yl)acrol­yl]amino}­benzoic acid; Geng *et al.*, 2013 ▸), but often associated with metal complex anions, *e.g.* the Cu^II^ complex with pyrophosphate, [Cu(H_2_O)(phen)(P_2_O_7_)]^2−^ (phen = 1,10-phenanthroline; Marino *et al.*, 2010 ▸), the mixed-valent di-Ru^II,III^ complex with 1-hy­droxy­ethane 1,1-di­phospho­nate (hedp) [Ru_2_(hedp)_2_ *X*]^4−^ (*X* = Cl, Br; Yi *et al.*, 2005 ▸) and the dioxo-Np complex anion salt with dipicolin­ate (dipic), [NpO_2_(dipic)(H_2_O)_2_]^−^ (Tian *et al.*, 2009 ▸).

## Synthesis and crystallization   

The title compound was synthesized by briefly heating together 0.5 mmol of 4-amino-3,5,6-tri­chloro­picolinic acid (picloram) with excess NaHCO_3_ in 10 ml of 10% (*v*/*v*) ethanol–water. Room temperature evaporation of the solution to dryness gave minor colourless crystal blocks of the title complex from which a specimen was cleaved for the X-ray analysis.

## Refinement details   

Crystal data, data collection and structure refinement details are summarized in Table 3 ▸. Hydrogen atoms of the water mol­ecules and the amine group were located in a difference-Fourier synthesis but were subsequently constrained in the refinement with the isotropic displacement parameters allowed to ride, with *U*_iso_(H) = 1.2*U*_eq_(N) or 1.5*U*_eq_(O).

## Supplementary Material

Crystal structure: contains datablock(s) global, I. DOI: 10.1107/S2056989015012633/wm5173sup1.cif

Structure factors: contains datablock(s) I. DOI: 10.1107/S2056989015012633/wm5173Isup2.hkl

CCDC reference: 1409779

Additional supporting information:  crystallographic information; 3D view; checkCIF report

## Figures and Tables

**Table 1:** Selected bond lengths ()

Na1O1*W* ^i^	2.4185(16)	Na1O2*W*	2.3183(17)
Na1O3*W* ^ii^	2.3803(16)	Na1O3*W*	2.3530(16)
Na1O1*W*	2.3529(16)		

Symmetry codes: (i) 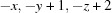; (ii) 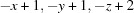.

**Table 2:** Hydrogen-bond geometry (, )

*D*H*A*	*D*H	H*A*	*D* *A*	*D*H*A*
O1*W*H11*W*O4*W*	0.82	1.97	2.786(2)	178
O1*W*H12*W*O6*W*	0.89	1.99	2.877(2)	174
O2*W*H21*W*O22^iii^	0.94	1.79	2.721(2)	171
O2*W*H22*W*O5*W*	0.84	1.98	2.816(2)	172
O3*W*H31*W*O5*W* ^iv^	0.88	1.93	2.781(2)	163
O3*W*H32*W*N1^ii^	0.90	2.02	2.910(2)	170
O4*W*H41*W*O22^v^	0.99	1.93	2.916(2)	173
O4*W*H42*W*O21	0.93	1.99	2.918(2)	174
O5*W*H51*W*O21	0.90	1.87	2.748(2)	166
O5*W*H52*W*O2*W* ^vi^	0.90	2.01	2.8481(19)	155
O6*W*H61*W*O22^vii^	0.93	2.10	2.972(2)	156
O6*W*H62*W*O21^iv^	0.93	2.19	2.996(2)	144
N4H41O6*W* ^viii^	0.96	2.18	3.080(2)	156
N4H42O4*W* ^viii^	0.97	2.22	3.146(2)	160

Symmetry codes: (ii) 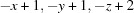; (iii) 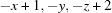; (iv) 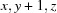; (v) 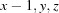; (vi) 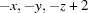; (vii) 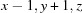; (viii) 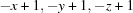.

**Table 3:** Experimental details

Crystal data	
Chemical formula	[Na(H_2_O)_3_](C_6_H_2_Cl_3_N_2_O_2_)3H_2_O
*M*_r_	371.53
Crystal system, space group	Triclinic, *P* 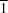
Temperature (K)	200
*a*, *b*, *c* ()	6.5625(5), 8.4574(6), 13.8553(10)
, , ()	78.747(6), 79.374(6), 88.864(6)
*V* (^3^)	741.17(9)
*Z*	2
Radiation type	Mo *K*
(mm^1^)	0.68
Crystal size (mm)	0.35 0.35 0.22

Data collection	
Diffractometer	Oxford Diffraction Gemini-S CCD detector
Absorption correction	Multi-scan (*CrysAlis PRO*; Agilent, 2014 ▸)
*T*_min_, *T*_max_	0.947, 0.980
No. of measured, independent and observed [*I* > 2(*I*)] reflections	6040, 2905, 2487
*R*_int_	0.025
(sin /)_max_ (^1^)	0.617

Refinement	
*R*[*F* ^2^ > 2(*F* ^2^)], *wR*(*F* ^2^), *S*	0.031, 0.088, 1.00
No. of reflections	2905
No. of parameters	182
H-atom treatment	H-atom parameters constrained
_max_, _min_ (e ^3^)	0.30, 0.28

Computer programs: *CrysAlis PRO* (Agilent, 2014 ▸), *SIR92* (Altomare *et al.*, 1993 ▸), *SHELXL97* (Sheldrick, 2008 ▸) within *WinGX* (Farrugia, 2012 ▸), *PLATON* (Spek, 2009 ▸).

## supplementary crystallographic information

### Crystal data

**Table:**

[Na(H_2_O)_3_](C_6_H_2_Cl_3_N_2_O_2_)·3H_2_O	*Z* = 2
*M**_r_* = 371.53	*F*(000) = 380
Triclinic, *P*1	*D*_x_ = 1.665 Mg m^−^^3^
Hall symbol: -P 1	Mo *K*α radiation, λ = 0.71073 Å
*a* = 6.5625 (5) Å	Cell parameters from 2548 reflections
*b* = 8.4574 (6) Å	θ = 3.8–28.8°
*c* = 13.8553 (10) Å	µ = 0.68 mm^−^^1^
α = 78.747 (6)°	*T* = 200 K
β = 79.374 (6)°	Block, colourless
γ = 88.864 (6)°	0.35 × 0.35 × 0.22 mm
*V* = 741.17 (9) Å^3^	

### Data collection

**Table:**

Oxford Diffraction Gemini-S CCD-detector diffractometer	2905 independent reflections
Radiation source: Enhance (Mo) X-ray source	2487 reflections with *I* > 2σ(*I*)
Graphite monochromator	*R*_int_ = 0.025
Detector resolution: 16.077 pixels mm^-1^	θ_max_ = 26.0°, θ_min_ = 3.3°
ω scans	*h* = −8→7
Absorption correction: multi-scan (*CrysAlis PRO*; Agilent, 2014)	*k* = −10→10
*T*_min_ = 0.947, *T*_max_ = 0.980	*l* = −17→13
6040 measured reflections	

### Refinement

**Table:**

Refinement on *F*^2^	Secondary atom site location: difference Fourier map
Least-squares matrix: full	Hydrogen site location: inferred from neighbouring sites
*R*[*F*^2^ > 2σ(*F*^2^)] = 0.031	H-atom parameters constrained
*wR*(*F*^2^) = 0.088	*w* = 1/[σ^2^(*F*_o_^2^) + (0.0478*P*)^2^ + 0.2264*P*] where *P* = (*F*_o_^2^ + 2*F*_c_^2^)/3
*S* = 1.00	(Δ/σ)_max_ = 0.001
2905 reflections	Δρ_max_ = 0.30 e Å^−^^3^
182 parameters	Δρ_min_ = −0.28 e Å^−^^3^
0 restraints	Extinction correction: *SHELXL97* (Sheldrick, 2008) within *WinGX* (Farrugia, 2012)
Primary atom site location: structure-invariant direct methods	Extinction coefficient: 0.050 (3)

### Special details

**Table:**

Geometry. Bond distances, angles *etc*. have been calculated using the rounded fractional coordinates. All su's are estimated from the variances of the (full) variance-covariance matrix. The cell e.s.d.'s are taken into account in the estimation of distances, angles and torsion angles
Refinement. Refinement of *F*^2^ against ALL reflections. The weighted *R*-factor *wR* and goodness of fit *S* are based on *F*^2^, conventional *R*-factors *R* are based on *F*, with *F* set to zero for negative *F*^2^. The threshold expression of *F*^2^ > σ(*F*^2^) is used only for calculating *R*-factors(gt) *etc*. and is not relevant to the choice of reflections for refinement. *R*-factors based on *F*^2^ are statistically about twice as large as those based on *F*, and *R*- factors based on ALL data will be even larger.

### Fractional atomic coordinates and isotropic or equivalent isotropic displacement parameters (Å^2^)

**Table:**

	*x*	*y*	*z*	*U*_iso_*/*U*_eq_	
Cl3	0.73580 (8)	0.09272 (5)	0.52593 (4)	0.0257 (2)	
Cl5	0.76678 (8)	0.74102 (5)	0.46993 (4)	0.0240 (2)	
Cl6	0.67863 (9)	0.70381 (6)	0.70472 (4)	0.0295 (2)	
O21	0.4767 (2)	0.04778 (17)	0.77535 (11)	0.0287 (5)	
O22	0.8188 (2)	0.04407 (17)	0.77067 (11)	0.0292 (5)	
N1	0.6684 (2)	0.39495 (18)	0.71307 (12)	0.0196 (5)	
N4	0.7849 (3)	0.42747 (19)	0.40294 (12)	0.0213 (5)	
C2	0.6814 (3)	0.2631 (2)	0.67196 (14)	0.0172 (5)	
C3	0.7193 (3)	0.2696 (2)	0.57064 (14)	0.0170 (5)	
C4	0.7473 (3)	0.4178 (2)	0.50250 (14)	0.0168 (6)	
C5	0.7335 (3)	0.5539 (2)	0.54712 (14)	0.0173 (5)	
C6	0.6946 (3)	0.5361 (2)	0.64975 (15)	0.0186 (6)	
C21	0.6561 (3)	0.1034 (2)	0.74534 (14)	0.0204 (6)	
Na1	0.24455 (12)	0.42819 (9)	1.01558 (6)	0.0268 (3)	
O1W	0.0817 (2)	0.53070 (17)	0.87914 (10)	0.0299 (5)	
O2W	0.1503 (2)	0.15728 (17)	1.05390 (11)	0.0300 (5)	
O3W	0.4202 (2)	0.62279 (17)	1.07140 (10)	0.0272 (5)	
O4W	0.1371 (2)	0.27585 (17)	0.77676 (12)	0.0317 (5)	
O5W	0.2700 (2)	−0.10534 (18)	0.95986 (11)	0.0325 (5)	
O6W	0.1709 (2)	0.82429 (17)	0.73270 (11)	0.0298 (5)	
H41	0.80460	0.32820	0.37840	0.0260*	
H42	0.80680	0.53330	0.36040	0.0260*	
H11W	0.09740	0.45450	0.85000	0.0450*	
H12W	0.10510	0.62480	0.83710	0.0450*	
H21W	0.15180	0.09520	1.11820	0.0450*	
H22W	0.19310	0.08550	1.02150	0.0450*	
H31W	0.38250	0.71930	1.04480	0.0410*	
H32W	0.40980	0.61800	1.13770	0.0410*	
H41W	0.02360	0.20490	0.77150	0.0480*	
H42W	0.25100	0.20920	0.77350	0.0480*	
H51W	0.34890	−0.07030	0.89970	0.0490*	
H52W	0.15590	−0.13130	0.93860	0.18 (2)*	
H61W	0.06160	0.89510	0.72480	0.0450*	
H62W	0.30130	0.87260	0.72460	0.0450*	

### Atomic displacement parameters (Å^2^)

**Table:**

	*U*^11^	*U*^22^	*U*^33^	*U*^12^	*U*^13^	*U*^23^
Cl3	0.0359 (3)	0.0159 (2)	0.0269 (3)	0.0007 (2)	−0.0044 (2)	−0.0091 (2)
Cl5	0.0280 (3)	0.0146 (2)	0.0282 (3)	−0.0001 (2)	−0.0049 (2)	−0.0012 (2)
Cl6	0.0412 (3)	0.0196 (3)	0.0309 (3)	0.0023 (2)	−0.0063 (2)	−0.0131 (2)
O21	0.0247 (8)	0.0260 (8)	0.0314 (9)	−0.0065 (6)	−0.0013 (6)	0.0013 (6)
O22	0.0290 (8)	0.0250 (8)	0.0318 (9)	0.0043 (6)	−0.0089 (7)	0.0013 (6)
N1	0.0202 (9)	0.0195 (8)	0.0195 (9)	0.0004 (6)	−0.0025 (7)	−0.0060 (7)
N4	0.0263 (9)	0.0197 (8)	0.0178 (8)	0.0009 (7)	−0.0031 (7)	−0.0042 (6)
C2	0.0128 (9)	0.0164 (9)	0.0220 (10)	0.0008 (7)	−0.0024 (7)	−0.0037 (7)
C3	0.0152 (9)	0.0147 (9)	0.0224 (10)	0.0005 (7)	−0.0038 (7)	−0.0063 (7)
C4	0.0106 (9)	0.0193 (10)	0.0216 (10)	0.0008 (7)	−0.0044 (7)	−0.0053 (8)
C5	0.0141 (9)	0.0144 (9)	0.0228 (10)	0.0002 (7)	−0.0041 (7)	−0.0019 (7)
C6	0.0160 (10)	0.0167 (9)	0.0252 (10)	0.0015 (7)	−0.0040 (8)	−0.0090 (8)
C21	0.0251 (11)	0.0192 (9)	0.0177 (10)	0.0014 (8)	−0.0028 (8)	−0.0068 (7)
Na1	0.0249 (5)	0.0264 (4)	0.0291 (5)	−0.0002 (3)	−0.0031 (3)	−0.0068 (3)
O1W	0.0360 (9)	0.0275 (8)	0.0252 (8)	0.0022 (6)	−0.0021 (7)	−0.0065 (6)
O2W	0.0390 (9)	0.0239 (8)	0.0246 (8)	0.0020 (6)	−0.0008 (7)	−0.0039 (6)
O3W	0.0322 (9)	0.0264 (8)	0.0223 (8)	0.0022 (6)	−0.0020 (6)	−0.0064 (6)
O4W	0.0282 (8)	0.0283 (8)	0.0399 (9)	0.0023 (6)	−0.0067 (7)	−0.0097 (7)
O5W	0.0342 (9)	0.0353 (9)	0.0247 (8)	0.0009 (7)	−0.0010 (7)	−0.0020 (7)
O6W	0.0257 (8)	0.0311 (8)	0.0330 (9)	−0.0003 (6)	−0.0043 (7)	−0.0079 (6)

### Geometric parameters (Å, º)

**Table:**

Na1—O1W^i^	2.4185 (16)	O4W—H42W	0.9300
Na1—O3W^ii^	2.3803 (16)	O4W—H41W	0.9900
Na1—O1W	2.3529 (16)	O5W—H52W	0.9000
Na1—O2W	2.3183 (17)	O5W—H51W	0.9000
Na1—O3W	2.3530 (16)	O6W—H61W	0.9300
Cl3—C3	1.7216 (18)	O6W—H62W	0.9300
Cl5—C5	1.7213 (18)	N1—C2	1.342 (2)
Cl6—C6	1.7289 (19)	N1—C6	1.330 (2)
O21—C21	1.243 (2)	N4—C4	1.342 (2)
O22—C21	1.250 (2)	N4—H41	0.9600
O1W—H12W	0.8900	N4—H42	0.9700
O1W—H11W	0.8200	C2—C3	1.370 (3)
O2W—H21W	0.9400	C2—C21	1.515 (3)
O2W—H22W	0.8400	C3—C4	1.407 (3)
O3W—H31W	0.8800	C4—C5	1.403 (2)
O3W—H32W	0.9000	C5—C6	1.376 (3)
			
O1W^i^—Na1—O3W^ii^	173.66 (6)	H41W—O4W—H42W	103.00
O1W—Na1—O3W	114.69 (6)	H51W—O5W—H52W	98.00
O1W—Na1—O1W^i^	86.32 (5)	H61W—O6W—H62W	116.00
O1W—Na1—O3W^ii^	100.02 (6)	C2—N1—C6	116.35 (16)
O2W—Na1—O3W	141.97 (6)	C4—N4—H41	118.00
O1W^i^—Na1—O2W	85.88 (6)	C4—N4—H42	118.00
O2W—Na1—O3W^ii^	92.71 (6)	H41—N4—H42	124.00
O1W—Na1—O2W	103.20 (6)	N1—C2—C3	123.14 (17)
O3W—Na1—O3W^ii^	84.24 (5)	N1—C2—C21	115.53 (16)
O1W^i^—Na1—O3W	93.06 (5)	C3—C2—C21	121.32 (16)
Na1—O1W—Na1^i^	93.68 (5)	Cl3—C3—C2	119.28 (14)
Na1—O3W—Na1^ii^	95.76 (6)	Cl3—C3—C4	119.39 (14)
Na1—O1W—H11W	100.00	C2—C3—C4	121.32 (16)
Na1^i^—O1W—H11W	121.00	N4—C4—C3	122.49 (16)
Na1—O1W—H12W	128.00	N4—C4—C5	122.95 (17)
H11W—O1W—H12W	112.00	C3—C4—C5	114.55 (17)
Na1^i^—O1W—H12W	103.00	Cl5—C5—C4	118.07 (14)
Na1—O2W—H22W	128.00	Cl5—C5—C6	121.71 (14)
H21W—O2W—H22W	97.00	C4—C5—C6	120.22 (16)
Na1—O2W—H21W	121.00	Cl6—C6—N1	115.37 (15)
Na1—O3W—H32W	119.00	Cl6—C6—C5	120.21 (14)
H31W—O3W—H32W	108.00	N1—C6—C5	124.42 (17)
Na1—O3W—H31W	109.00	O21—C21—O22	127.24 (18)
Na1^ii^—O3W—H32W	115.00	O21—C21—C2	116.83 (17)
Na1^ii^—O3W—H31W	109.00	O22—C21—C2	115.91 (17)
			
O3W^ii^—Na1—O3W—Na1^ii^	0.00 (6)	N1—C2—C21—O21	89.1 (2)
O1W—Na1—O1W^i^—Na1^i^	0.00 (6)	N1—C2—C3—Cl3	179.26 (15)
O2W—Na1—O1W^i^—Na1^i^	−103.53 (6)	N1—C2—C3—C4	0.0 (3)
O3W—Na1—O1W^i^—Na1^i^	114.57 (6)	C21—C2—C3—Cl3	0.7 (3)
O1W—Na1—O3W^ii^—Na1^ii^	114.11 (6)	C3—C2—C21—O22	89.4 (2)
O2W—Na1—O3W^ii^—Na1^ii^	−141.98 (6)	N1—C2—C21—O22	−89.3 (2)
O3W—Na1—O3W^ii^—Na1^ii^	−0.02 (8)	C3—C2—C21—O21	−92.2 (2)
O2W—Na1—O3W—Na1^ii^	87.03 (10)	C2—C3—C4—C5	0.1 (3)
O1W^i^—Na1—O3W—Na1^ii^	174.25 (5)	Cl3—C3—C4—N4	0.6 (3)
O2W—Na1—O1W—Na1^i^	84.89 (6)	Cl3—C3—C4—C5	−179.13 (15)
O3W—Na1—O1W—Na1^i^	−91.68 (6)	C2—C3—C4—N4	179.9 (2)
O1W^i^—Na1—O1W—Na1^i^	0.00 (5)	C3—C4—C5—Cl5	179.71 (15)
O3W^ii^—Na1—O1W—Na1^i^	−179.91 (5)	N4—C4—C5—Cl5	−0.1 (3)
O1W—Na1—O3W—Na1^ii^	−98.40 (6)	N4—C4—C5—C6	−180.0 (2)
C6—N1—C2—C21	178.63 (17)	C3—C4—C5—C6	−0.2 (3)
C2—N1—C6—Cl6	−179.62 (14)	C4—C5—C6—N1	0.2 (3)
C6—N1—C2—C3	0.0 (3)	Cl5—C5—C6—Cl6	−0.2 (3)
C2—N1—C6—C5	−0.1 (3)	Cl5—C5—C6—N1	−179.72 (15)
C21—C2—C3—C4	−178.59 (18)	C4—C5—C6—Cl6	179.74 (16)

Symmetry codes: (i) −*x*, −*y*+1, −*z*+2; (ii) −*x*+1, −*y*+1, −*z*+2.

### Hydrogen-bond geometry (Å, º)

**Table:**

*D*—H···*A*	*D*—H	H···*A*	*D*···*A*	*D*—H···*A*
O1*W*—H11*W*···O4*W*	0.82	1.97	2.786 (2)	178
O1*W*—H12*W*···O6*W*	0.89	1.99	2.877 (2)	174
O2*W*—H21*W*···O22^iii^	0.94	1.79	2.721 (2)	171
O2*W*—H22*W*···O5*W*	0.84	1.98	2.816 (2)	172
O3*W*—H31*W*···O5*W*^iv^	0.88	1.93	2.781 (2)	163
O3*W*—H32*W*···N1^ii^	0.90	2.02	2.910 (2)	170
O4*W*—H41*W*···O22^v^	0.99	1.93	2.916 (2)	173
O4*W*—H42*W*···O21	0.93	1.99	2.918 (2)	174
O5*W*—H51*W*···O21	0.90	1.87	2.748 (2)	166
O5*W*—H52*W*···O2*W*^vi^	0.90	2.01	2.8481 (19)	155
O6*W*—H61*W*···O22^vii^	0.93	2.10	2.972 (2)	156
O6*W*—H62*W*···Cl6	0.93	2.83	3.4400 (15)	124
O6*W*—H62*W*···O21^iv^	0.93	2.19	2.996 (2)	144
N4—H41···Cl3	0.96	2.54	2.9956 (17)	109
N4—H41···O6*W*^viii^	0.96	2.18	3.080 (2)	156
N4—H42···Cl5	0.97	2.52	2.9690 (17)	108
N4—H42···O4*W*^viii^	0.97	2.22	3.146 (2)	160

Symmetry codes: (ii) −*x*+1, −*y*+1, −*z*+2; (iii) −*x*+1, −*y*, −*z*+2; (iv) *x*, *y*+1, *z*; (v) *x*−1, *y*, *z*; (vi) −*x*, −*y*, −*z*+2; (vii) *x*−1, *y*+1, *z*; (viii) −*x*+1, −*y*+1, −*z*+1.
